# Chromatin structure is distinct between coding and non-coding single nucleotide polymorphisms

**DOI:** 10.1186/1471-2199-15-22

**Published:** 2014-10-05

**Authors:** Hongde Liu, Jinchen Zhai, Kun Luo, Lingjie Liu

**Affiliations:** 1 State Key Laboratory of Bioelectronics, Southeast University, Nanjing 210096, China; 2 Department of Neurosurgery, Xinjiang Evidence-Based Medicine Research Institute, The First Affiliated Hospital of Xinjiang Medical University, Urumqi 830054, China

**Keywords:** Single nucleotide polymorphism (SNP), Nucleosome, Histone modification, DNA methylation, Mutation

## Abstract

**Background:**

Previous studies suggested that nucleosomes are enriched with single nucleotide polymorphisms (SNPs) in humans and that the occurrence of mutations is closely associated with CpG dinucleotides. We aimed to determine if the chromatin organization is genomic locus specific around SNPs, and if newly occurring mutations are associated with SNPs.

**Results:**

Here, we classified SNPs according their loci and investigated chromatin organization in both CD4^+^ T cell and lymphoblastoid cell in humans. We calculated the SNP frequency around somatic mutations. The results indicated that nucleosome occupancy is different around SNPs sites in different genomic loci. Coding SNPs are mainly enriched at nucleosomes and associated with repressed histone modifications (HMs) and DNA methylation. Contrastingly, intron SNPs occur in nucleosome-depleted regions and lack HMs. Interestingly, risk-associated non-coding SNPs are also enriched at nucleosomes with HMs but associated with low GC-content and low DNA methylation level. The base-transversion allele frequency is significantly low in coding-synonymous SNPs (P < 10^-11^). Another finding is that at the -1 and +1 positions relative to the somatic mutation sites, the SNP frequency was significantly higher (P < 3.2 × 10^-5^).

**Conclusions:**

The results suggested chromatin structure is different around coding SNPs and non-coding SNPs. New mutations tend to occur at the -1 and +1 position immediately near the SNPs.

## Background

Single nucleotide polymorphisms (SNPs) are most common type of DNA sequence variation identified in individual genomes and are used as markers to identify disease-associated genes. Previous studies showed that chromatin structure affects SNP distribution [1-3]. Nucleosome, a structure of 146-bp DNA double helix wrapped on surface of a histone core, is the basic repeat unit of chromatin. Generally, nucleosomes are depleted near transcription start sites (TSSs) and are phased as a nucleosome arrays downstream of TSSs [4]. Higasa et al. revealed a periodicity of 146 nucleotides of SNP density around TSSs in CpG islands-associated genomic region; the periodical patterns suggested the location of nucleosomes that are phased at TSSs [2]. In Japanese killifish, it was found that point mutations occur approximately at nucleosomes and that insertions and deletions longer than 1 bp are present in nucleosome-depleted regions (NDRs) [1]. This was confirmed in the human genome in our laboratory [5]. Further analysis in human cells showed that, although SNPs are enriched in the core region of bulk nucleosomes, they are not enriched in nucleosomes containing the H2A.Z variant or histone H3 tri-methylated at lysine 4 (H3K4me3) [3].

Providing a polymorphism arises as a result of a mutation [6], a SNP results from a single base mutation that substitutes one nucleotide for another. CpG dinucleotides mutate at a high rate of C- > T because cytosine is vulnerable to deamination [7]. Chen et al suggested that nucleosomal DNA undergoes fewer C- > T mutations because of suppressed cytosine hydrolytic deamination relative to nucleosome-depleted DNA [8]. Selection appears to be acting on particular base substitutions to maintain optimum GC compositions in nucleosome and linker regions [9]. Non-CpG mutation rates are lowest in the open chromatin regions of the genome [10].

Despite the advancement in this field, the following open questions remain. First, the literature suggested that most SNPs sites are enriched at nucleosomal DNA; we asked if this enrichment is genomic locus specific, and if SNPs are associated with specific HMs. Second, what is the difference between risk-associated SNPs and neutral SNPs in terms of histones modifications, nucleosome occupancy and GC-content? Third, SNPs are the results of ancient mutations. Does newly occurring mutation associate with a SNP?

In this work, we investigated the distributions of nucleosomes, histone modification, DNA methylation and GC-content around human SNPs sites and revealed that coding-SNPs are enriched at nucleosomes, whereas intron-SNPs are mostly in nucleosome-depleted regions. DNA methylation and repressed HMs marked around the coding-SNPs. The base-transversion allele frequency was significantly lower in coding-synonymous SNPs than in Risk SNPs. In cancer genomics, somatic mutation frequency was significantly higher at the -1 and +1 positions relative to SNP sites. Our results show some detailed characteristics of SNPs from an epigenetics perspective.

## Results

### Nucleosome occupancy is distinct at SNPs sites in different genomic loci

**Figure 1:**
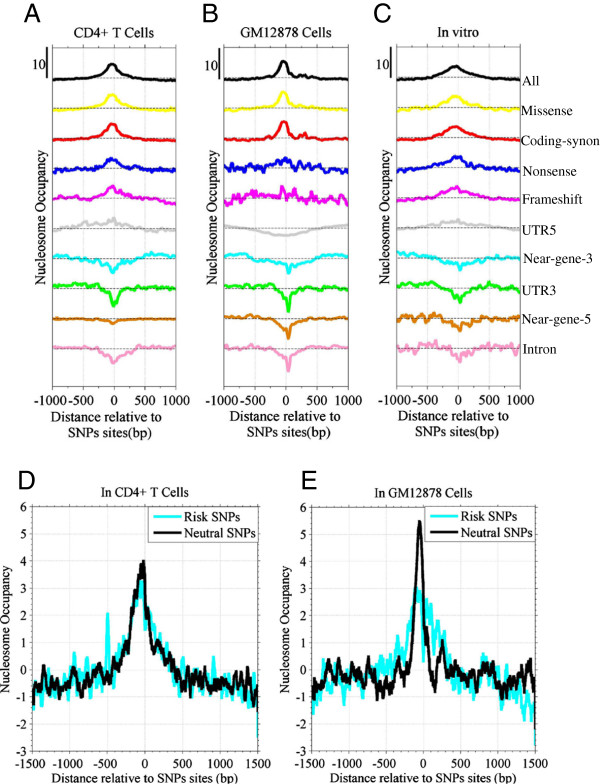
**Coding-SNPs are enriched at nucleosomes and intron SNPs are in nucleosome-depleted regions. A**, **B** and **C**: Nucleosome occupancy around 2 kbp of nine categories of SNPs in CD4^+^ T cells **(A)**, GM12878 cells **(B)** and *in vitro***(C)**, respectively; **D**: Nucleosome occupancy around both risk and neutral SNPs sites in CD4^+^ T cells; **E**: Same as D but in GM12878 cells.

The literature suggested that SNPs are enriched at bulk nucleosomes, but are depleted at H2A.Z-containing nucleosomes or nucleosomes with H3K4me3 [3]. Here, we showed that the nucleosome organization is genomic locus-specific around SNPs sites. According to the annotation of dbSNP build 130 from the UCSC genome browser, human SNPs are classified into nine types (Additional file 1: Figure S1A-B). We calculated the nucleosome occupancy profiles for the nine types of SNPs in CD4^+^ T cells, lymphoblastoid cells (GM 12878) and *in vitro*. The distribution of SNPs shows a periodic pattern near TSSs (Additional file 1: Figure S1C). Although the nucleosome occupancy profile peaks at overall SNPs (Figure 1), it differentially positions at the nine types of SNP sites. SNPs in coding regions (coding SNPs) are consistently enriched in nucleosomal DNA in three states of cells (Figure 1A-C). Contrastingly, intron SNPs were mostly in NDRs, regardless of the state of the cells (Figure 1A-C). The SNPs in 3′-untranslated regions (UTR3) were also in NDRs. Most SNPs in the 5′ and 3′ regions of genes are actually in intergenic regions, they are also enriched at NDRs. The SNPs in 5′-untranslated regions (UTR5) are nucleosome-occupied in CD4^+^ T cells and *in vitro*, but are nucleosome-depleted in the lymphoblastoid cells. In order to confirm that the patterns of nucleosome occupancy are not due to random factor, we randomly selected genomic sites in exon, intron, UTR5, UTR3 and 5′ and 3′ of genes, and calculated profiles of nucleosomes around the random sites. Result indicated that the profiles do not exhibit obvious patterns (Additional file 1: Figure S2), suggesting the patterns of nucleosome occupancy shown in Figure 1 are significant.

Taken together, the results indicated that nucleosome organization is distinct between coding-SNPs and noncoding SNPs.

It was observed that the patterns of nucleosome occupancy were almost consistent between *in vivo* (Figure 1A-B) and *in vitro* (Figure 1C) conditions, suggesting that the DNA sequence has a role in patterns of SNPs relative to nucleosomes. In addition, the peak of nucleosome occupancy at coding SNPs is wider *in vitro* than *in vivo* (Figure 1A-C), probably suggesting a fuzzy nucleosome *in vitro*.

We then compared nucleosome occupancy for risk SNPs and neutral SNPs (coding-synonymous SNPs). In both CD4^+^ T cells and lymphoblastoid cells, risk SNPs are enriched at nucleosomes (Figure 1D and E). In fact, risk SNPs contain only a small faction of coding-SNPs (exon SNP = 13.3%) (Additional file 1: Figure S1D-E). Thus, according to the results shown in Figure 1A-C, risk SNPs should be mainly in NDRs; however, they are at nucleosome, indicating a specific feature.

### Repressive HMs are enriched at coding-synonymous SNPs sites and depleted at intron-SNP sites

**Figure 2:**
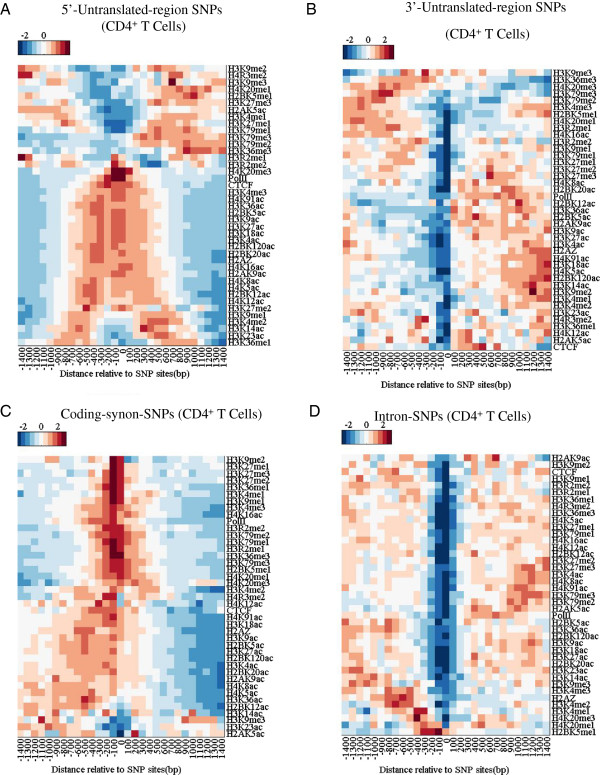
**Coding SNPs are encompassed within repressed histones modifications and intron SNPs lack histone modifications. A**, **B**, **C** and **D**: Distributions of histone methylations and acetylations around 5′-untranslated region (UTR5)-SNPs sites **(A)**, 3′-untranslated region (UTR5)-SNPs sites **(B)**, coding-synonymy SNPs sites **(C)** and intron-SNPs sites **(D)**, respectively.

We calculated the profiles of the HMs around SNPs sites in CD4^+^ T cells and lymphoblastoid cells. Profiles of HMs around 5′-untranslated SNPs (UTR5 SNPs) sites are typically consistent with the distributions of HMs at promoters and gene bodies (Figure 2A) [11]. The literature suggested that activation modifications, including acetylations of histones and H3K4me3, cover at the promoter and the 5′ end of genes, and repressive modifications (such as H3K27me3 are H3K9me3) are lacking in these regions [11]. In our results, around UTR5-SNPs, most acetylations are enriched and the repressive methylations are depleted (Figure 2A). Around UTR3 SNPs, most of HMs is depleted (Figure 2B).

Importantly, we observed an enrichment of repressed HMs near coding-synonymous SNPs (Figure 2C) and a depletion of HMs near intron SNPs (Figure 2D). Considering the results in Figure 1A, this suggested that those nucleosomes around coding-synonymous SNPs sites are marked with repressive HMs. By contrast, intron-SNPs lack HMs because they are located in NDRs (or linker DNA). Some types of HMs are conserved between CD4^+^ T cell and lymphoblastoid cell (Additional file 1: Figure S3A-D and Figure S4), such as H3K79me2 for UTR5 SNPs, H3K79me3 for coding-synonymous SNPs, H3K36me3 for intron SNPs. However, some of them vary greatly, such as H3K27me3 for coding-synonymous SNPs (Additional file 1: Figure S4).

The difference in HMs between coding-synonymous and intron SNPs can be attributed to specific distribution of HMs among genome. Generally, nucleosome occupancy is higher in exons than in introns [4]. Due to this, as expected, HMs will be more enrichment in exons than in introns. Thus, we observed the HMs around SNPs show a more enrichment in coding regions than in introns.

### Risk non-coding SNPs are enriched at nucleosomes with histone modifications

**Figure 3:**
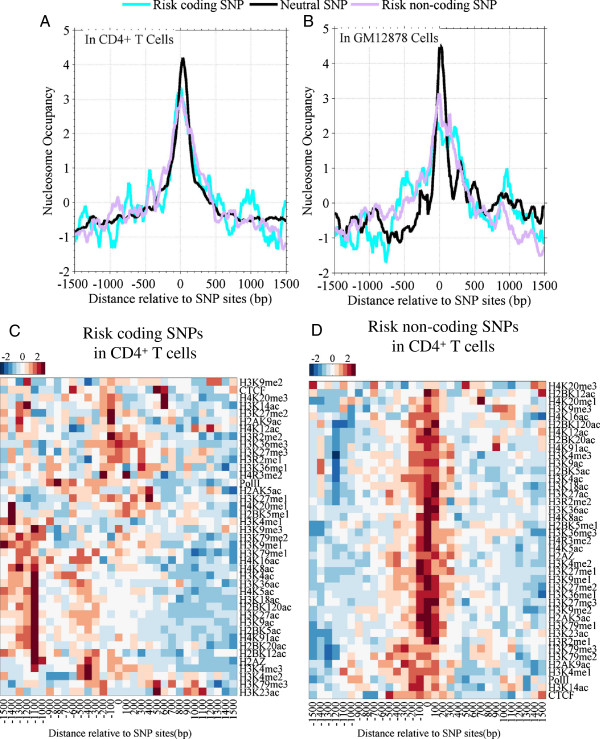
**Both coding and non-coding risk SNPs are enriched at nucleosomes and the risk non-coding SNPS associate HMs. A** and **B**, profiles of nucleosome occupancy around neutral SNPs, risk coding SNPs and risk non-coding SNPs in both CD4^+^ T cells **(A)** and GM12878 cells **(B)**; **C** and **D**, profiles of epigenetic marks (HMs, Pol II, H2A.Z and CTCF) around risk coding SNPs **(C)** and around risk non-coding SNPs **(D)**.

In Figure 1, we observed coding-SNPs are enriched at nucleosomes and intron-SNPs are depleted at nucleosomes. Interestingly, although risk SNPs contain only a small faction (13.3%) of coding-SNPs, nucleosome occupancy is high around the risk SNPs. To this end, we then tested the nucleosome occupancy and HMs for risk coding-SNPs and risk non-coding SNPs, respectively. Results suggested that both coding and non-coding risk SNPs are enriched at nucleosomes in CD4^+^ T cells (Figure 3A-B), although nucleosome occupancy around the risk SNPs sites is less than that around neutral SNPs sites. Profiles of HMs around risk coding-SNPs sites are similar to those around coding-synonymous SNPs (neutral SNPs) sites (Figures 3C and 2C). That is, around the type of SNPs sites, acetylated modifications are depleted and repressed HMs are enriched. Importantly, we found that around the risk non-coding SNPs, both repressed and transcription-activated HMs are enriched (Figure 3D), suggesting a distinct characteristic. Also, histone acetyltransferases (HATs) (CBP, P300, PCAF and MOF) and histone deacetylase (HDAC6) are enriched around risk SNPs and depleted around neutral SNPs (Additional file 1: Figure S3E-F).

We tested whether HMs can discriminate the risk SNPs and neutral SNPs. We identified those HMs that are different between the two types of SNPs (Additional file 1: Figure S5A and Table S1) and constructed linear classifiers (Additional file 1: Figure S5B). The classifier with four types of HMs achieves an AUC of 0.69 in the ROC curve (Additional file 1: Figure S5B), suggesting that HMs discriminately mark the genomic loci around the risk and the neutral SNPs.

### GC content and DNA methylation level are low near risk SNP sites

**Figure 4:**
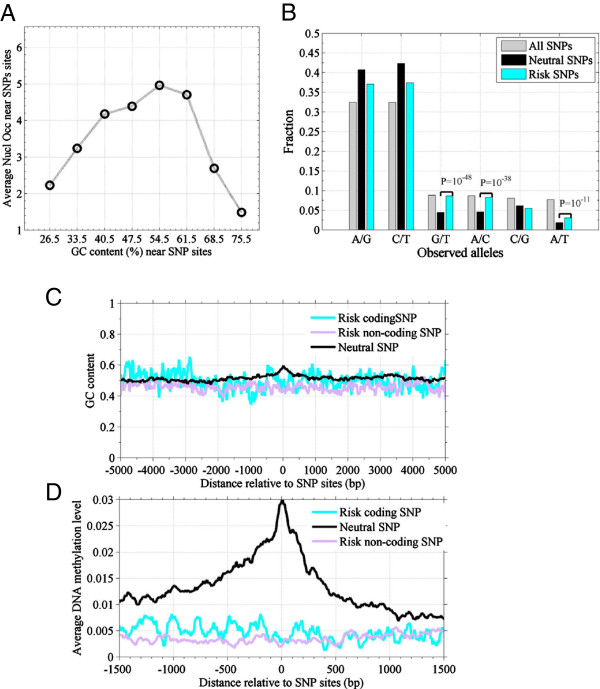
**Coding-synonymous SNPs (neutral SNPs) have a low frequency of base-transversion alleles.** Risk SNPs exhibit a low GC-content background and are associated with a low DNA methylation level, while neutral SNPs associate with high GC-content and high DNA methylation. **A**: Average nucleosome occupancy against GC-content around SNPs sites; **B**: Observed allele frequencies of SNPs in humans. Neutral SNPs refer to coding-synonymous SNPs. The different significant P-values are indicated. Base transition refers to A/G and C/T substitution; base transversion refers to G/T, A/C, C/G and A/T. The Base transition is three-fold higher than that of base transversion in SNPs; **C**: GC-content profile around neutral SNPs and risk SNPs sites; **D**: DNA methylation levels around neutral SNPs and risk SNPs sites.

SNPs result from a single base mutation [6]. CpG dinucleotides show a high rate of C- > T mutations [7], and non-CpG mutation rates are lowest in open chromatin [10]. SNPs are tightly correlated with both base substitution and GC-content.Here, we firstly plotted average nucleosome occupancy against GC-content around SNPs sites (Figure 4A). Nucleosome occupancy near SNPs sites is highest when GC-content is near 54%. With an increase or a decrease of GC-contents, nucleosome occupancy decreases around SNPs.

We then investigated the allele frequency for SNPs. As expected, the frequency of base transitions (A/G and C/T) is three-fold higher than that of base transversions (G/T, A/C, C/G and A/T) in SNPs (Figure 4B). Interestingly, the base-transversion frequency of neutral SNPs is significantly lower than that of risk SNPs (P < 10^-11^) (Figure 4B).

The GC-content profile is significantly higher around neutral SNPs sites than that around risk SNPs sites (P = 2.1 × 10^-9^ (*t*-test)) (Figure 4C). In fact, GC-content profile goes uniformity around risk SNPs sites, but it greatly increases near neutral SNPs sites (Figure 4C). The GC-content profiles around the nine types of SNPs indicated that coding SNPs are associated with a higher GC-content than non-coding SNPs (Additional file 1: Figure S6C-D). Moreover, DNA methylation level is also much higher near neutral SNPs sites than that near risk SNPs sites (Figure 4D). Around the risk coding SNPs, both GC content and DNA methylation show a higher level than those around the risk non-coding SNPs (Figure 4C and D), but the differences are not significant (P = 0.07 for GC content; and P = 0.11 for DNA methylation (*t*-test)). We also found the nucleosomes are positioned at base-transition risk SNPs, but are depleted at base-transversion risk SNPs (Additional file 1: Figure S6C).

### New mutations occur frequently at -1 and +1 base position around SNPs sites

**Figure 5:**
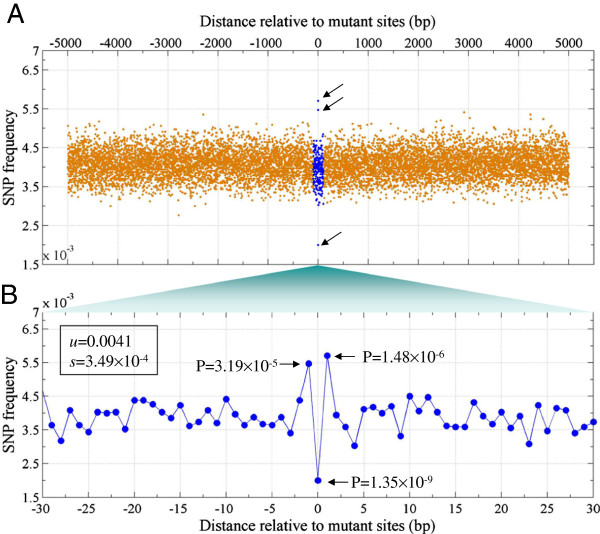
**New mutations occur at SNP sites with a lower probability and frequently occur at the -1 and +1 position relative to the mutation sites. A**: SNP frequency around the somatic mutation sites of malignant melanoma; **B**: A zoom-in plot of Figure 4A. P-values are calculated from normalization distribution of SNP frequencies.

Considering the close relationship between SNPs and mutations, we asked if new somatic mutations are associated with SNPs. Using a dataset of somatic mutation sites from malignant melanoma [12], we calculated the SNP frequency of a 10-kbp region around the somatic mutation sites. The results showed that SNP frequency distribution is stable distal the mutation sites (Figure 5A). However, at the -1 and +1 position relative to the mutation sites, the SNP frequency is significantly (1.4-fold) higher (P <3.2 × 10^-5^, Z-test (*u* = 0.0041; *s* = 3.49 × 10^-4^)) (Figure 5B). This suggests that new mutations tend to occur at the -1 and +1 position proximal to SNPs sites (Figure 5B). Moreover, the SNP frequency is 2-fold lower at the mutation sites (P = 1.35 × 10^-9^) (Figure 5B). This indicates that a new mutation occurs at a SNP site with a lower probability; i.e. mutation of a SNP occurs at a low frequency.

## Discussion

SNP distribution shows an imprint of nucleosome positions in genomes [1,3]. SNP is caused by a single base mutation and CpG dinucleotides is highly correlated with mutation [7,9,10]. In this work, we investigated the chromatin organization around SNPs and analyzed the association between SNP allele frequency, mutation and GC-content using genome-wide dataset of nucleosomes and HMs in the human genome.

Firstly, we revealed that the nucleosome organization is genomic locus-specific around SNPs. Whether a SNP occurs at a nucleosome is determined by the genomic locus where it is in. We revealed that coding SNPs are located at nucleosomes, which is consistent with previous studies [3]. However, intron SNPs occur in nucleosome depleted regions (Figure 1A-C). This represents new knowledge concerning nucleosomes and SNPs. The specific selection of SNP for nucleosomes is probably due to consequence of interplay between nucleosomes and DNA sequence variation in evolution, particularly in introns [13]. Moreover, the characteristics are conserved in CD4^+^ T cells, lymphoblastoid cells and *in vitro* (Figure 1), indicating a certain stability of the epigenetic state around SNPs and also a tight association between genetic variation and chromatin structure in evolution.

Tolstorukov et al. suggested that SNPs are depleted at H3K4me3- or H2A.Z-containing nucleosomes [3]. We found that coding SNPs are enriched at nucleosomes and are associated both repressive HMs and DNA methylation in CD4^+^ T cell (Figures 2C and 4D). Given the stability of the epigenetic state across cell types and SNP arises from a mutation, our results suggested heritable mutations tend to occur at heterochromatin.

Secondly, risk non-coding SNPs are enriched at the nucleosomes with HMs. Risk coding SNPs show similar features to coding-synonymous SNPs. They are enriched at nucleosomes marked with both repressed HMs and high DNA methylation (Figure 3A-C). This suggests that the “risk” of such type of SNPs is due to their capacity of changing the type of amino acid in expression. However, risk non-coding SNPs show a specific feature. They are at nucleosomes and are associated with high a level of HMs, a low DNA methylation and an enrichment of both acetyltransferases and deacetylase (Figures 3D, 4D and Additional file 1: Figure S3 E-F).

Taken together, the specific chromatin structure suggested that risk non-coding SNPs occur probably in functional DNA regions. In transcription and DNA duplication, those functional regions will be bound by transcription factor (TF) and other proteins. This type of SNPs will alter interaction between DNA and proteins, thus leads to a deleterious consequence [14,15]. Gaffney et al indicated that SNPs could affect the positioning of nucleosome arrays by changing TF binding affinity in humans [16].Thirdly, we suggested that new mutations occur at a SNP sites with a low probability. However, they tend to occur at the -1 and +1 position relative to SNP sites (Figure 5B). The association between SNPs and new mutations is interesting, and provides a clue to identify mutations in cancer cells.

Regardless of some new findings, we should address two limitations in the study. One is about the data of mutation sites. The data of mutation sites is from human cancer cell [12]. Due to genetic instability and specific cell state, both mutation rate and mutation mechanism are probably distinct between cancer cells and normal cells. Thus, the finding regarding the distribution of SNP and mutation sites is only confined in the cancer cells. The other is about the choosing for neutral SNPs. We selected coding-synonymous SNPs as neutral SNPs. In recent literature [17], it was suggested that although coding-synonymous variation don not alter the sequence of the encoded protein, they can alter exonic splicing motifs and affect mRNA splicing in oncogenes in cancer cells. Thus, our definition for neutral SNP has a flaw.

## Conclusion

In this work, we investigated the association between SNPs and chromatin organization. The results indicated chromatin structure is distinct between coding SNPs and non-coding SNPs. Coding SNPs are enriched at nucleosomes, and are seemingly marked with repressive histone modifications. Non-coding SNPs are mainly at nucleosome-depleted regions with less histone modifications. Risk SNPs also occur in nucleosome and are associated with low levels of DNA methylation and specific histone modifications. Also, new mutations tend to occur at the -1 and +1 base position relative to SNP sites. Regardless of limitations in data selection, our analysis sheds light on the interplay between genetic variation and chromatin state.

## Methods

### Coordinates of SNPs sites and TSSs

Genomic coordinates of both SNPs sites and TSSs were retrieved from UCSC using the Tables function (http://genome.ucsc.edu). We limited “Class of variant” of SNP to be “single” to ensure the data only contains single-base variation. SNPs are classified into nine categories according to UCSC annotation. Only validated SNPs were used in further analysis. Coding-synonymous SNPs were chosen as neutral SNPs. Risk-associated SNPs (risk SNP) data were retrieved from (http://www.genome.gov/gwastudies/) [18]. We further divided risk SNPs into risk SNPs in coding region (risk coding SNPs) and risk SNPs in non-coding SNPs region (risk non-coding SNPs).

### Dataset of nucleosomes and histone modifications

For resting CD4^+^ T cells, the coordinates of ChIP-Seq tags of 20 histone methylations, one histone variant H2A.Z, Pol II [19], 18 histone acetylations [11] and nucleosomes [20] were retrieved. Data of the DNA methylation levels (the mean methylation percentages) at promoters (-1 kbp to +0.6 kbp relative to TSS) and gene bodies were taken from GEO of NCBI (GSM871287), which is determined in naïve CD4^+^ T Cells of patients with psoriasis or atopic dermatitis [21]. Data of the binding locations of histone acetyltransferases (HATs) (CBP, P300, PCAF and MOF) and histone deacetylase (HDAC6) were retrieved from the literature (GSE15735) [22].

Sequencing datasets of nucleosomes in a lymphoblastoid cell line (GM12878) and *in vitro* were retrieved from the literature (GSE35586) [23]. ChIP-seq data of 10 histone modifications, one histone variant H2A.Z and CTCF in the GM12878 cells [24], were downloaded from the UCSC ftp server (http://genome.ucsc.edu). A dataset of somatic mutation sites from cancer genomics of malignant melanoma was retrieved from the literature [12].

The genomic coordinates including nucleosomes, histone modifications, DNA methylation, binding sites of HATs, SNP sites, mutation sites and TSSs are extracted according to the hg18 human assembly.

### Profiles of histones modifications, DNA methylation, nucleosome positioning, Pol II, H2AZ and CTCF around SNPs

For histones modifications, Pol II, H2AZ and CTCF, the number of sequencing tags was counted at every genomic location in a 3 k-bp region surrounding the SNPs on positive and negative strands, respectively, thus resulting in two profiles. The two profiles were then aligned by oppositely moving with an appropriate shift [25]. The two profiles were then summed at every corresponding location to generate a final profile; then the profile were divided by average tags coverage of whole genome. In comparing, the profile was divided by amount of SNPs.

For nucleosome tags, the coordinates of the tags were extended to 146 bp in the 3′ direction prior to profiling and the shift was limited at 73 bp [25]. To test GC content effect on nucleosome occupancy, GC content of a 600-bp genomic region surrounding each SNP was calculated; and average of nucleosome occupancy profile of a 200-bp genomic region (length of nucleosomal DNA + linker DNA) near the SNP was calculated; then the average of nucleosome occupancy was plotted against GC content for all of SNPs.

For HATs (CBP, P300, PCAF and MOF) and HDAC6, their binding profiles were similarly generated around SNPs using the coordinates of bound regions identified in literature [21]. Profile of DNA methylation was calculated simply by summating the mean methylation percentages around SNPs, then the profile was averaged by dividing number of SNPs.

We also randomly selected genomic sites in exon, intron, UTR5, UTR3 and 5′ and 3′ of genes, and calculated profiles of nucleosomes around the random sites, respectively. The number of the random loci is equal to that of the corresponding type of SNPs.

### Identification of differential HMs between neutral SNPs and risk SNPs

Two-sample *t*-tests were used to test capacity of each type of HMs in distinguishing neutral SNPs and risk SNPs. First, HM’s level was calculated around 200 bp of both neutral SNPs and risk SNPs. Then different significance (P-value) of the HM levels between the two types of SNPs is tested with *t*-tests. Receiver operating characteristic (ROC) curve was also employed. The neutral SNPs and the risk SNPs were randomly mixed and divided into ten portions. Seven portions of the data were used as training data and three portions were as test data. Linear classifiers were constructed with each of HMs types. Each of classifier was represented as a parameter set of a constant (forced as 1) and slopes. Output of the classifier on the test data was then used to plot ROC curve.

### Density profiles of SNPs in the vicinity of mutant sites

Density profile of SNPs was calculated by counting the total number at every genomic site in a 10 k-bp region around mutant sites. A Z-test was used to calculate the different significant P-values of SNP density at each genomic locus.

### Observed allele frequencies

Frequencies of alleles was counted for six types of base substitution (base transitions: A/G and C/T; base transversions: G/T, A/C, C/G and A/T) by checking SNP’s annotation. We obtained the fraction of each allele for neutral SNPs, risk SNPs and all of SNPs, respectively. Difference of the fraction of each allele between the neutral SNPs and the risk SNP was tested using a Z-test.

## Abbreviations

SNP: Single nucleotide polymorphism; HM: Histone modification; TSS: Transcription start sites; NDR: Nucleosome-depleted region; Neutral SNP: Coding-synonymous SNPs; Risk SNP: Risk-associated SNPs.

## Competing interests

The authors declare that they have no competing interests.

## Authors’ contributions

LHD designed the work and prepared the manuscript. LK, ZJC and LLJ performed data analysis. All authors read and approved the final manuscript.

## Supplementary Material

Additional file 1: Figure S1 A: Nine categories of SNPs; B: Number of SNPs; C: SNP frequencies; D: Number of risk-associated SNPs (risk SNPs) [18]; E: Distribution of risk SNPs. **Figure S2.** Profiles of nucleosome occupancy around random genomic loci. **Figure S3.** A-D: Profiles of histones modifications near SNPs sites in lymphoblastoid cell (GM12878 cells); E-F: Binding of histone acetylases and deacetylase at neutral and risk SNPs sites in CD4^+^ T cells. **Figure S4.** Correlation coefficients of profiles of HMs, H2AZ and CTCF between CD4^+^ T cells and lymphoblastoid cells (GM12878 cells). **Figure S5.** HMs can be used to distinguish risk SNPs and neutral SNPs. A: Difference significance; B: Receiver operating characteristic (ROC) curves of the linear classifier models. **Figure S6.** A: GC-content profiles around SNPs; B: Average GC-content around SNPs; C: Nucleosome occupancy profiles for both base transition (A/G and C/T) and base transversion (G/T, A/C, C/G and A/T). Click here for file
