# Supplementary material for: Chromatin structure is distinct between coding and non-coding single nucleotide polymorphisms
Source: BMC Mol Biol. 2014 Oct 5;15:22. doi: 10.1186/1471-2199-15-22 (PMC4193957; doi:10.1186/1471-2199-15-22)

Supplementary Materials

Chromatin structure is distinct between coding and non-coding single nucleotide polymorphisms

Hongde Liu[1]*; Jingchen Zhai[1]; Kun Luo[2]; Lingjie Liu[1]

[1]State Key Laboratory of Bioelectronics, Southeast University, Nanjing 210096, China
[2]Department of Neurosurgery, Xinjiang Evidence-Based Medicine Research Institute, The First Affiliated Hospital of Xinjiang Medical University, Urumqi 830054, China

Table S1

Linear classifier parameters (Col.2) and discriminating capacity (Col.3) of each of HMs

	Parameters in the classifier	-log10(P-value) of a single HM
Const	1.0	
H3K36me3	0.052	189
H4K20me1	-0.130	159
H3K9me2	-0.108	132
H4K16ac	-1.398	129
H2BK5me1	0.395	124
H3K79me1	-0.164	82
H3R2me1	-0.819	75
H3K27me3	-4.089	65
PolII	-0.356	54
H3K9me3	0.433	53
H3K4me3	-0.768	44
H4K91ac	-2.121	44
H3K9me1	-0.0142	42
H3K27me1	-0.714	30
H3K27me2	-0.711	25
H3K23ac	1.514	20
H3K14ac	-5.423	19
H2BK120ac	0.254	17
H3K4me2	-0.228	16
H2BK5ac	-1.599	16
H3K4me1	0.00146	14
H3K79me3	-0.0648	14
H3K4ac	0.130	14
H3K27ac	0.331	13
H3K9ac	-0.0319	12
H4K12ac	-1.0626	12
H2BK20ac	2.370	11
H3R2me2	0.321	11
H4K8ac	-1.139	9
H2AK9ac	-5.144	9
H3K18ac	0.273	9
H4K20me3	-1.204	4
H4K5ac	-1.278	4
H3K36me1	0.866	3
H2AZ	3.246	1.5
H4R3me2	0.0722	1.2
H3K79me2	-1.237	1.255
H2BK12ac	0.100	1.0
H2AK5ac	0.364	1.0
CTCF	2.240	1.0
H3K36ac	-2.539	0.03

Figure S1

A: Scheme indicating genomic regions for nine categories of SNPs;
B: Number of SNPs in the nine categories of SNPs;
C: SNP frequencies around transcription start sites (TSSs);
D: Number of risk-associated SNPs (risk SNPs); the risk SNPs data were retrieved from (http://www.genome.gov/gwastudies/) (Hindorff et al., 2009);
E: Distribution of risk SNPs in different genomic regions.

A

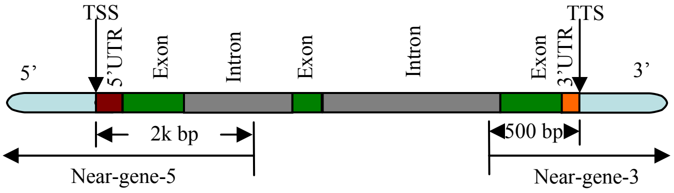

B

Number of nine types of SNPs

Types of SNPs	Number	Total
Intron	4707162	4707162
Near-gene-5	218436	
Near-gene-3	50817	
UTR5	20648	204944
UTR3	89937	
Frameshift	538	
*Coding-synon	45601	
Missense	47557	
Nonsense	663	

* Randomly selected 14508 coding-synonymous SNPs as neutral SNPs

C

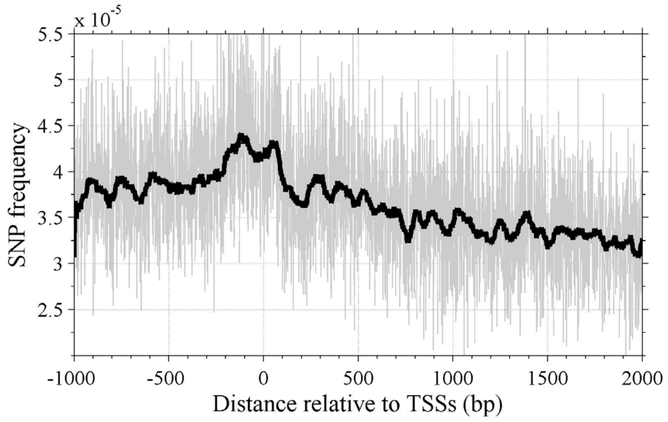

D

Distribution of risk SNPs in different genomic loci

Distribution	Number
Exon	1898
Intron	6721
Inter-gene	2237
UTR5 & UTR3	3652
Total	14508

E

Percentage of risk SNPs in different genomic loci

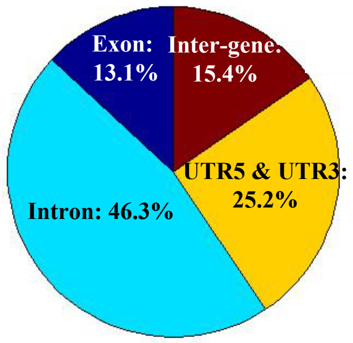

Figure S2

Profiles of nucleosome occupancy around random genomic loci.
Random genomic loci are selected in exon, intron, UTR5, UTR3 and 5' and 3' of genes. Profile of nucleosome occupancy is calculated around the random loci, respectively.

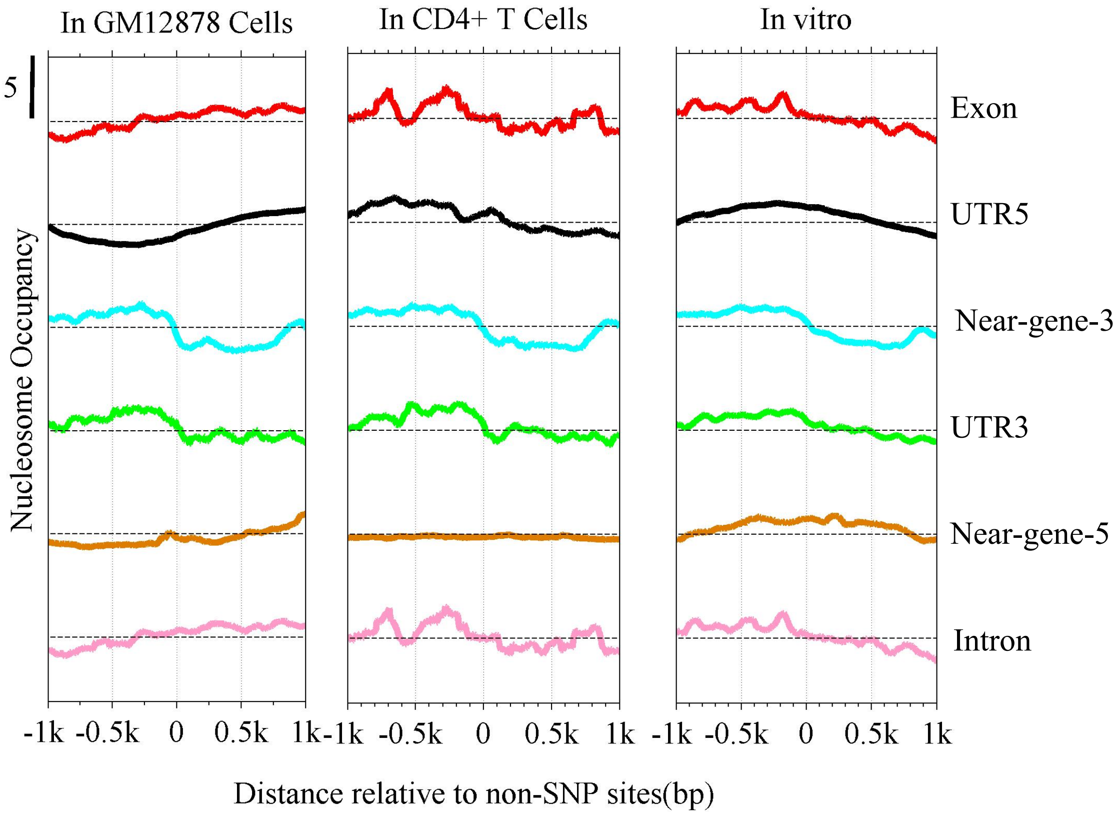

Figure S3

Profiles of histones modifications near SNPs sites in lymphoblastoid cell (GM12878 cells).
A-D: Histones methylations near around 5'-untranslated region (UTR5)-SNPs sites (A), 3'-untranslated region (UTR5)-SNPs sites (B), coding-synonymous SNPs sites (C) and intron-SNPs sites (D), respectively, in lymphoblastoid cells;
E and F: Binding of histone acetylases and deacetylase at neutral SNPs sites (E) and risk SNPs sites (F), respectively, in CD4+ T cells.

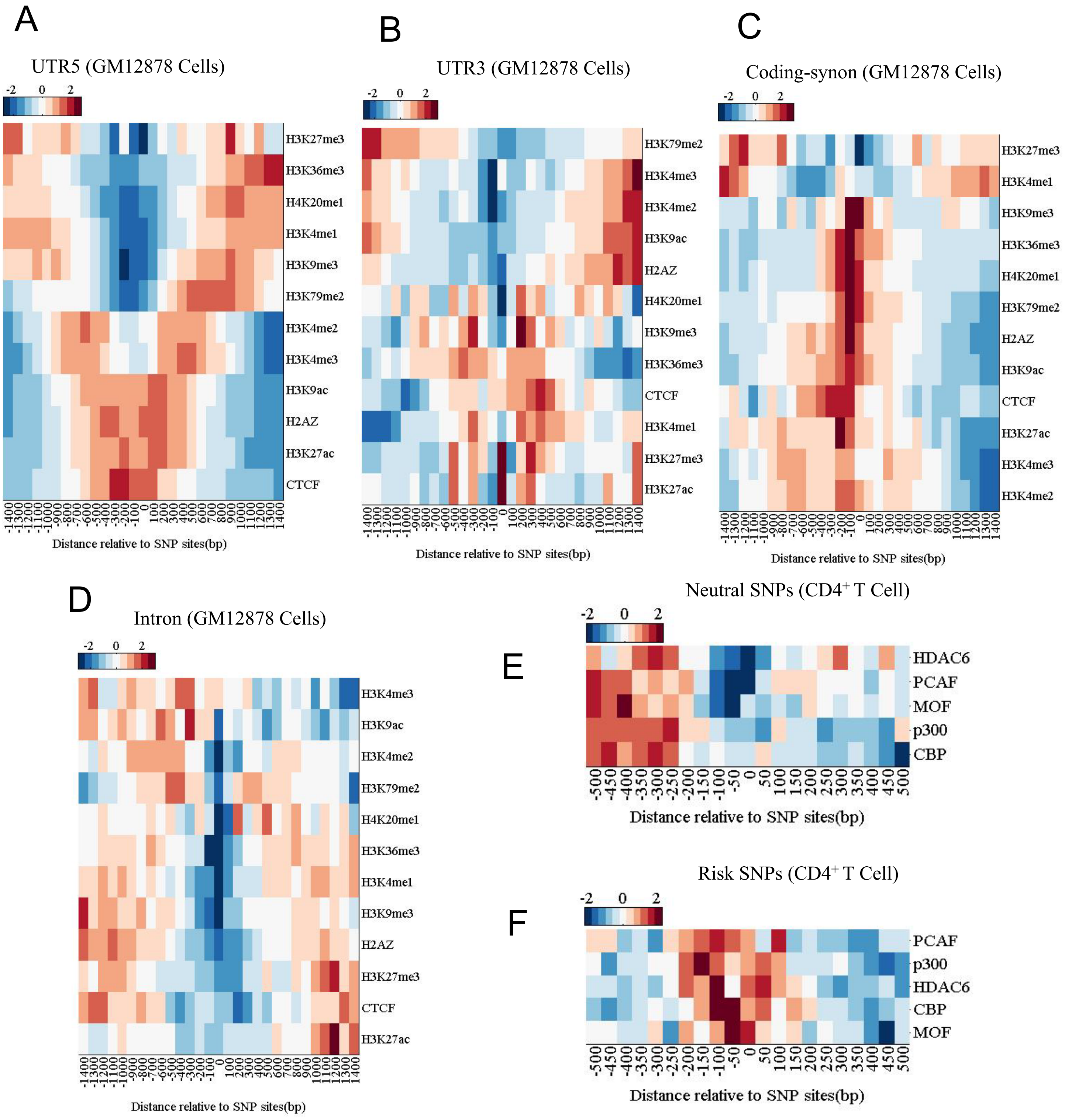

Figure S4

Correlation coefficients of profiles of HMs, H2AZ and CTCF between CD4+ T cells and lymphoblastoid cells (GM12878 cells) .

The profile is for 3000-bp genomic region around SNPs. The calculation is for the four types of SNPs, UTR5-SNPs, UTR3-SNPs, coding-synonymous SNPs and intron-SNPs

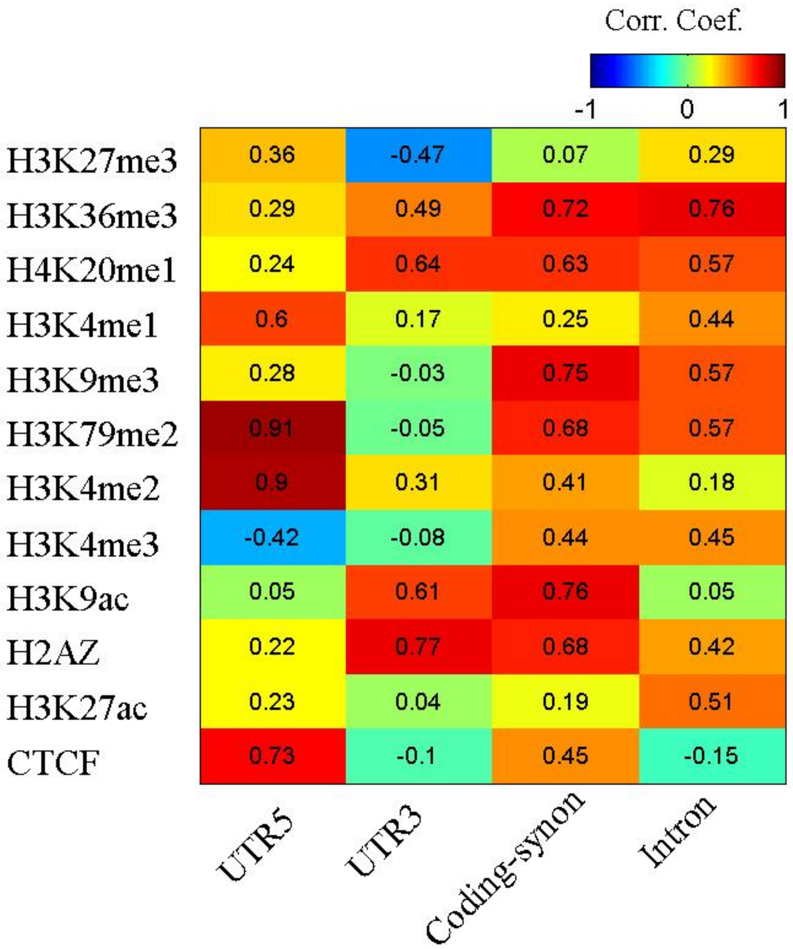

Figure S5

Histones modifications can be used to distinguish risk SNPs and neutral SNPs.

A: Histones modifications that are different between risk SNPs and neutral SNPs; P-values that indicate difference significance were calculated with a two-sample *t*-test;

B: Receiver operating characteristic (ROC) curves of the linear classifier models that identify risk SNPs. The linear classifier parameters are listed in Table S1. Features refer to chromatin marks. The features are sorted according to the different significant P-values (Table S1). The 4, 8, 16 and all features were chosen, respectively, to construct the models. Area under the ROC curve (AUC) is indicated for each of the models.

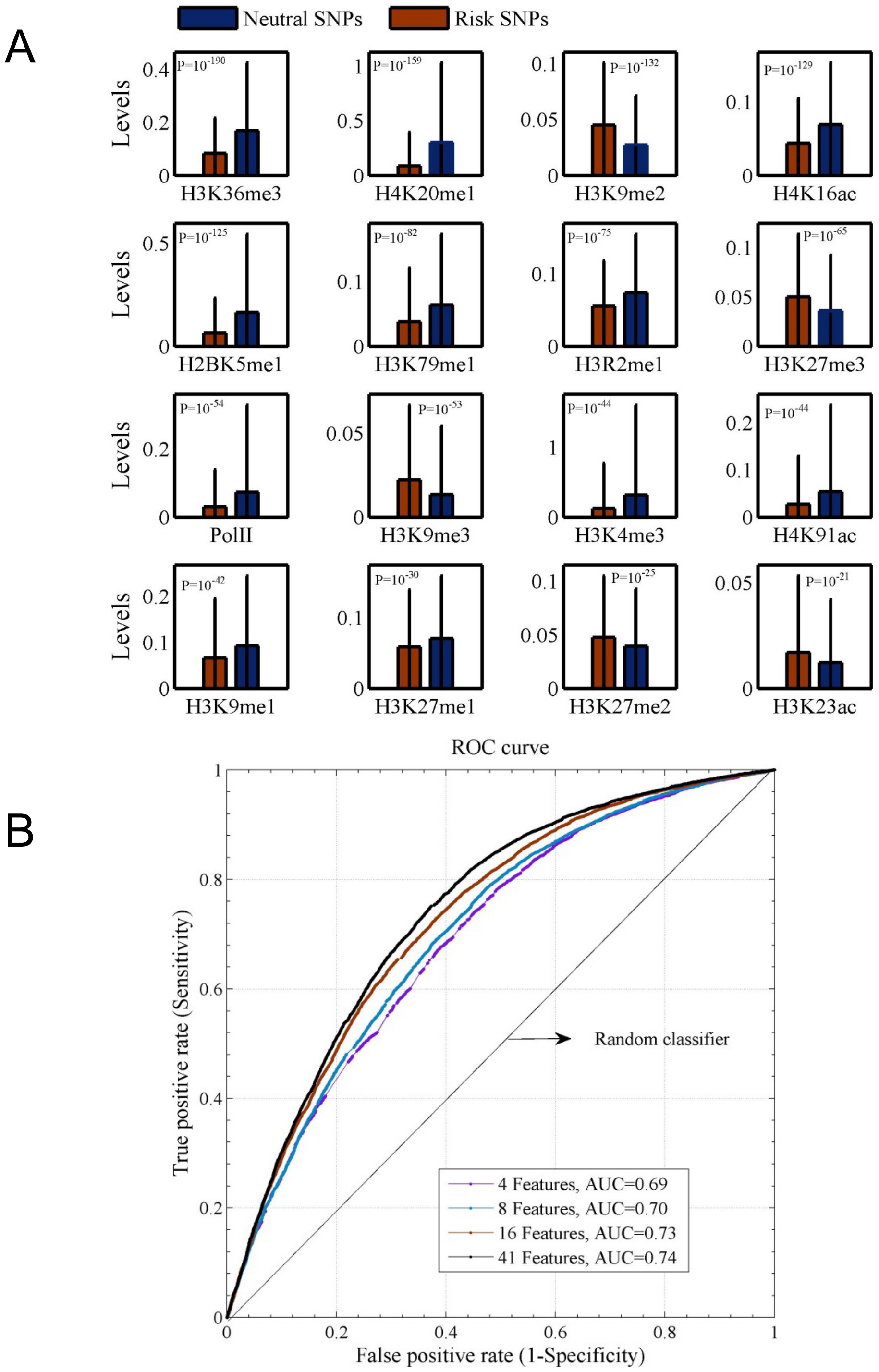

Figure S6

A: GC-content profiles around nine categories of SNPs;

B: Average GC-content in a 2-kbp region around the nine categories of SNPs;

C: Nucleosome occupancy profiles for both base transition (A/G and C/T) and base transversion (G/T, A/C, C/G and A/T) in both risk SNPs and neutral SNPs.

A

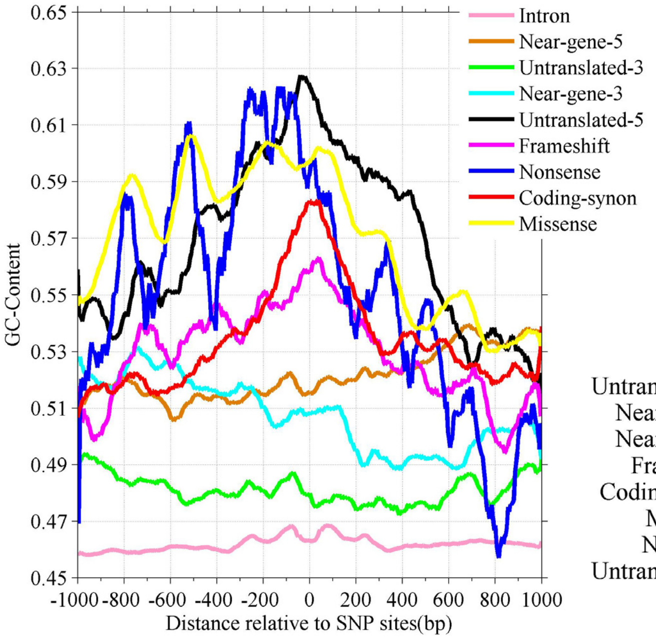

B

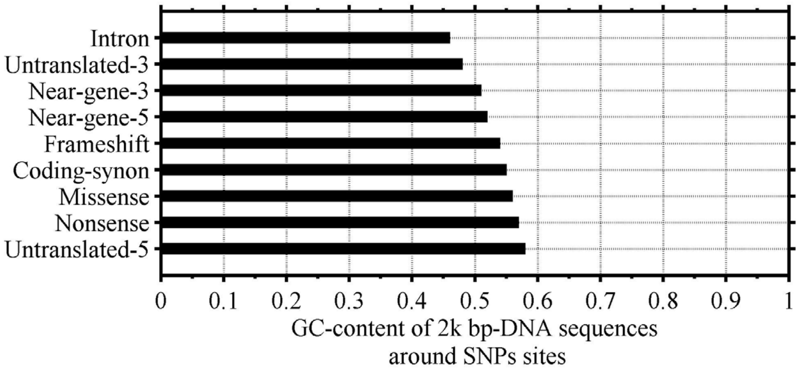

C

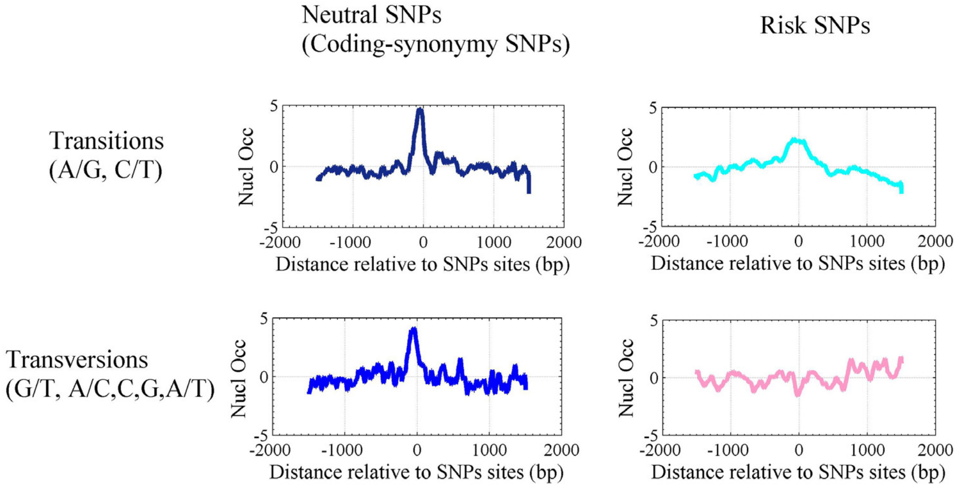

Supplement: Additional file 1: Figure S1 — A: Nine categories of SNPs; B: Number of SNPs; C: SNP frequencies; D: Number of risk-associated SNPs (risk SNPs) [18]; E: Distribution of risk SNPs. Figure S2. Profiles of nucleosome occupancy around random genomic loci. Figure S3. A-D: Profiles of histones modifications near SNPs sites in lymphoblastoid cell (GM12878 cells); E-F: Binding of histone acetylases and deacetylase at neutral and risk SNPs sites in CD4+ T cells. Figure S4. Correlation coefficients of profiles of HMs, H2AZ and CTCF between CD4+ T cells and lymphoblastoid cells (GM12878 cells). Figure S5. HMs can be used to distinguish risk SNPs and neutral SNPs. A: Difference significance; B: Receiver operating characteristic (ROC) curves of the linear classifier models. Figure S6. A: GC-content profiles around SNPs; B: Average GC-content around SNPs; C: Nucleosome occupancy profiles for both base transition (A/G and C/T) and base transversion (G/T, A/C, C/G and A/T). [file 1471-2199-15-22-S1.pdf]
